# Implementation of Regular Lifestyle Counseling During Long-Term Follow-Up Care of Childhood Cancer Survivors: Monocentric Prospective Study

**DOI:** 10.2196/59614

**Published:** 2024-12-26

**Authors:** Franziska Richter, Lea Louisa Kronziel, Inke König, Thorsten Langer, Judith Gebauer

**Affiliations:** 1 Department of Pediatric Oncology and Hematology, University Medical Center Schleswig-Holstein, Lübeck, Germany; 2 Institute of Medical Biometry and Statistics, University of Luebeck, Lübeck, Germany; 3 Department of Internal Medicine I, University Medical Center Schleswig-Holstein, Lübeck, Germany

**Keywords:** lifestyle counseling, long-term follow-up, childhood cancer survivors, physical activity, metabolic disorders, cancer survivor, treatment-related, risk of obesity, metabolic syndrome, healthy lifestyle, morbidity, patient, hypercholesterolemia, diabetes mellitus, health care professionals

## Abstract

**Background:**

Many childhood cancer survivors (CCS) develop treatment-related late effects, including an increased risk of obesity and metabolic syndrome. A healthy lifestyle can reduce the risk of associated comorbidities. Therefore, at-risk CCS could benefit from lifestyle counseling during regular long-term follow-up (LTFU).

**Objective:**

We implemented a new form of care to decrease the long-term morbidity among CCS and to gain new insights into the lifestyle of those patients.

**Methods:**

Over a 1-year study period, lifestyle counseling was integrated into LTFU care. Metabolic disorders, including hypercholesterolemia, diabetes mellitus, overweight or underweight, and low activity levels, were assessed as screening parameters for various risk groups. The perspectives of CCS, physicians, and sports scientists were compared to identify those with the highest needs. Each lifestyle counseling included general recommendations for physical activity, as well as an assessment of individual preferences for and barriers to the implementation of a healthy lifestyle. A follow-up appointment after 1 month was performed.

**Results:**

Of the 155 CCS aged 18 to 63 years (n=100, 65% female and n=55, 35% male), 112 (72%) had an indication for lifestyle counseling, identified by physicians, sports scientists, or the CCS themselves. Metabolic disorders affected 45% (n=70) of these CCS, and 46% (n=72) did not meet recommended activity levels. A total of 120 (77%) CCS received lifestyle counseling, including 8 initially uninterested individuals who became open to recommendations. Those with intensive cancer treatment history showed the greatest need. A total of 65 (54%) CCS were advised to change their lifestyle in both areas (diet and exercise) while 51 (43%) CCS received recommendations for only exercise (n=43 CCS, 36%) or diet (n=8 CCS, 7%). A total of 4 (3%) CCS, although interested in counseling, received no advice, as they already met the recommendations. Follow-up revealed high adherence to recommendations and successful integration into daily lives. In total, 97% (n=150) of survivors indicated that the provision of lifestyle counseling during LTFU would be generally beneficial.

**Conclusions:**

Incorporating specialized health care professionals such as sports scientists into survivorship care enhances the multidisciplinary approach of LTFU care. Promoting a healthy lifestyle by offering guideline-based lifestyle counseling is broadly accepted among CCS and may reduce long-term morbidity.

## Introduction

Due to improved treatment modalities, the proportion of patients surviving childhood and adolescent cancer has been steadily increasing in recent decades, and 5-year overall survival now exceeds 85% in German childhood cancer survivors (CCS) [1]. Currently, more than 40,000 former patients are included in the long-term follow-up (LTFU) cohort of the German Childhood Cancer Registry [2]. These CCS were diagnosed with cancer at least 5 years ago, so regular oncological follow-up has usually already been completed. During their lifetime, many of these survivors are affected by further health problems and diseases caused by the cancer and its therapy, so-called late effects [3]. These can manifest in different organs of the entire body. Due to hormonal changes and altered body composition that may occur during or after therapy, the risk of obesity and associated diseases such as diabetes mellitus, cardiovascular disease, and hypertension increases [4]. Cardiovascular disease, in turn, is among the most common nonmalignant causes of death in survivors of cancer, with some groups such as Hodgkin lymphoma survivors having a particularly high risk [5,6]. Long-term sequelae may not only manifest in physical form; many CCS develop psychological problems, particularly depression or fatigue syndrome, during or even years after cessation of cancer treatment [7,8]. For these CCS at risk, it is particularly important to develop interventions or treatments that can positively influence their long-term health.

According to Smith et al [9], over 70% of CCS do not meet the lifestyle guidelines of the World Cancer Research Fund. Furthermore, in more than 30% of the adult CCS (median age: 32.7 years, 1598 participants), metabolic syndrome was already prevalent, which is a combination of metabolic parameters associated with increased cardiovascular risk. Consequently, multiple studies evaluated the effect of counseling on CCS’ lifestyle and could demonstrate a reduction in comorbidities, as well as a better quality of life due to a healthy lifestyle [10,11]. Furthermore, blood pressure in CCS improved due to lifestyle counseling in previous studies [12]. Therefore, a healthier lifestyle could result in less comorbidities including metabolic syndrome among CCS [10]. A healthy lifestyle in this context includes in general no smoking, the awareness of alcohol consumption, and regular physical activity, as well as a vitamin and nutrition-rich diet [13].

Additionally, previous studies demonstrated that the occurrence of late effects can be positively influenced by regular physical activity, exercise, and a healthy diet [14,15]. Especially avoiding overweight and obesity has a significant impact on the severity of late effects associated with the metabolic syndrome [11]. According to Hammoud et al [16], despite the available guidelines, many cardiometabolic risk factors in CCS remain underdiagnosed or undertreated although CCS have a 4-fold increased risk of cardiac-related mortality compared to the general population. The authors, therefore, recommend further studies that incorporate nutrition, as well as physical activity, taking into account the heterogeneity of late effects and the impact of cancer treatment on the individual’s physical abilities. Both the type and intensity of exercise may differ from the type of sport practiced before the disease, but physical activity, in general, has a benefit [15] as it not only improves body composition and reduces the risk of future health conditions, it also often positively affects mental health [17]. Additional factors such as age, gender, and socioeconomic status (that correlate inversely with the risk of obesity) also need to be considered in lifestyle counseling [18-20]. As interest in lifestyle counseling may decrease with increasing time interval from diagnosis and late effects often do not manifest until adulthood, it is especially important to inform and counsel children, adolescents, and young adults about the importance of prevention in terms of a healthy lifestyle [21]. The earlier the education about a healthy lifestyle including physical activity and healthy nutrition starts, the more likely this behavior will be maintained in future life [22]. Therefore, the implementation of regular lifestyle counseling within the framework of LTFU care could be a useful and practical approach to informing CCS about a healthy lifestyle in order to prevent future diseases.

In 2014, an interdisciplinary LTFU clinic for CCS (“LTFU clinic”) was established at the University Medical Center Schleswig-Holstein, Campus Luebeck [23]. Among others, we were a consortium partner in the multicenter study CARE for CAYA that took place from 2018 to 2021 and provided specialized lifestyle counseling for high-risk CCS [24]. Based on these experiences, we decided to implement lifestyle counseling as part of standard care for every CCS during their regular visit to our LTFU clinic. It included general, guideline-based recommendations, as well as individualized content based on the survivor’s needs. Risk factors for cardiometabolic diseases such as cancer treatment, age, gender, and socioeconomic status were assessed to identify high-risk CCS. Additionally, we performed an analysis to determine how many CCS are in need of lifestyle counseling according to current guidelines [11,13] due to pre-existing metabolic diseases and how they accepted and implemented counseling into their daily life. A subgroup analysis was conducted to identify CCS who may escape standard inclusion criteria for lifestyle counseling. Furthermore, we aimed to develop and establish a lifestyle counseling program considering the physician’s, the sports scientist’s, and CCS’ perspective on who could benefit most from counseling that, due to limited resources, would be mainly limited to at-risk CCS in most settings.

## Methods

### Study Population

In this study, lifestyle counseling as a routine care offer was implemented within regular LTFU care. All CCS visiting our late effects clinic from February 2022 to the end of January 2023 were offered to participate in lifestyle counseling. They were informed about this offer several weeks before their visit to the LTFU clinic by phone and asked to participate in this study during their stay in the clinic. Survivors who were 18 years or older at initial cancer diagnosis but had undergone cancer treatment in our local department for pediatric oncology, mainly young adults with medulloblastoma, were also transitioned to our late effects clinic and thus analyzed as part of the study group. Risk stratification into three different risk groups, based on survivors’ initial cancer diagnosis and treatment, their risk for late effects, and their need for LTFU examinations, was performed according to Gebauer et al [25], with risk group 1 corresponding to a low, group 2 to a medium, and group 3 to a high risk for late effects.

### Inclusion Criteria

Inclusion criteria were hypercholesterolemia (total cholesterol ≥5.0 mmol/L), diabetes mellitus (preexisting or hemoglobin A_1c_ [HbA_1c_] ≥6.5%), BMI ≤18.5 or ≥30 kg/m², not reaching the recommended activity time (<150 minutes moderate or <75 minutes vigorous activity time per week), or the need for counseling expressed by the survivor [11].

These inclusion criteria were based on three different perspectives on who would benefit most from counseling: the perspective of the survivor (expresses the need for counseling), the perspective of the sports scientist (focus on activity level and anthropometric data), and the perspective of the treating physician (focus on metabolic diseases).

### Exclusion Criteria

CCS who were not interested in lifestyle counseling or study participation were excluded from this study.

### Assessment of Inclusion Criteria

HbA_1c_ was assessed in order to check if CCS were in a diabetic or prediabetic condition (HbA_1c_ level between 5.7% and 6.4%) [26]. For BMI calculation, most CCS were measured wearing clothes, as well as with their prosthesis, if needed. Only one CCS came to the LTFU care without a prosthesis, therefore, we used a formula to correct the BMI for CCS with amputated limbs [27]. Additionally, we calculated the BMI with the Amputee Coalition BMI Calculator Widget.

### Ethical Considerations

This study was approved by the ethics committee of the University of Luebeck (registration 18‐087) and conducted in accordance with the Helsinki Declaration of 1975. Informed consent was obtained from all participants included in the study, with the ability to opt-out. Data were saved anonymously. Patients took part in the study voluntarily and received no compensation other than counseling.

### Lifestyle Counseling

Every counseling was performed by the same sports scientist (FR) with several years of experience in counseling of CCS. It was scheduled for 30 minutes and took place in the LTFU clinic right after the regular LTFU visit. The CCS were informed about the appointment before they attended their appointment in the LTFU clinic. Medical and anthropometric data was documented in the clinic’s database. Additionally, CCS were asked to answer a set of lifestyle questions (physical activity time, daily activity, and nutritional status) to evaluate to which extent the lifestyle recommendations from the American College of Sports Medicine and the American Cancer Society (ASC) were already implemented [11]. Due to those guidelines, cancer survivors should be physically active for at least 150 minutes per week with moderate intensity or 75 minutes of vigorous-intensity. Physical activity should be performed for at least 10 minutes at a time, and sedentary activities should be avoided or reduced. It is also recommended that adults perform strength exercises at least twice a week for the major muscle groups [11,28]. Every CCS was asked to assess their subjective requirement for the need for nutrition or sports counseling. Sports behavior, as well as nutrition behavior, was assessed by subjective questioning.

During counseling, the sports scientist referred to the recommendations of the ASC and German Nutrition Society, considering the third, updated, and evidence-based ASC nutrition and physical activity guideline for cancer survivors that was published early during the study period in 2022 [11,29]. In addition to pointing out general recommendations, the sports scientist emphasized individual aspects that were not in concordance with the guidelines. Consequently, although every CCS was asked the same set of questions, the content of lifestyle counseling depended on the CCS’s individual needs (Multimedia Appendix 1). If CCS already met the recommendations for physical activity and were still interested in the counseling, counseling focused on general lifestyle advices according to the guidelines from Rock et al [11] such as smoking cessation and nutrition counseling.

After 4 weeks, every CCS received a remote follow-up appointment, to check whether they were able to implement the recommendations in their daily life and whether they benefited from the counseling (Multimedia Appendix 1). All statements were answered subjectively.

### Statistical Analysis

For descriptive statistics, the median and range were calculated for continuous variables. For categorical variables, the absolute number, as well as the relative number of the respective category, is presented.

Qualitative data were analyzed descriptively. They were only collected for one question in a structured follow-up interview by phone (“effect of lifestyle counseling”). The first author (FR) made field notes during the interview. Afterward, the perceived effect was categorized into three different subcategories (weight loss, quality of life including more joy, balance, and satisfaction, or both). These categories were determined after obtaining an overview of patients’ responses. This approach was supervised by JG. Reliability was tested by asking patients about their current weight and comparing this answer to the documented weight in the database.

The programming language R (version 4.2.2; R Core Team) was used for the entire analysis of the data.

### Subgroup Analysis

For further analysis, three subgroup analyses were defined to gain a better overview of CCS who may escape standard inclusion criteria for lifestyle counseling. Subgroup 1 was considered to include the CCS who had a need for counseling based only on the criteria of the sports scientist and not from the physician’s point of view. Subgroup 2 included CCS who requested lifestyle counseling but did not need it based on the inclusion criteria. Subgroup 3 included CCS who met the inclusion criteria but rejected the offer for counseling. We descriptively compared the subgroups regarding relevant characteristics using the chi-square test or Fisher exact test where appropriate.

## Results

### Survivors’ Characteristics

Overall, lifestyle counseling was offered to 155 CCS (n=100, 65% female and n=55, 35% male), who had a median age of 30 years (IQR 24-39.5) with a range of 18 to 63 years. Most CCS underlying diseases were lymphoma (n=45, 29%), leukemia (n=42, 27%), and brain tumors (34/155, 22%). The median age at first diagnosis was 12 years (IQR 5-16) with a range of 0 to 35 years. In terms of diagnosis and therapy, 18 (12%) CCS were assigned to risk group 1, 42 (27%) CCS to risk group 2, and 95 (61%) CCS to risk group 3. A total of 61% (11/18) of survivors assigned to risk group 1 had an indication for lifestyle counseling and 45% (19/42) of those assigned to risk group 2. In the group with the highest risk for late effects (risk group 3), 72% (68/95) had need for lifestyle counseling. Detailed information on the characteristics of the CCS is shown in Table 1. In addition, 29 (19%) survivors were in a prediabetic condition, of which 10 (6%) survivors were not included in the needs analysis as they did not meet any inclusion criteria. Furthermore, 82 (53%) of all CCS already received previous nutrition or sports counseling, either organized by themselves, during rehabilitation stays, or while being part of the study CARE for CAYA [21].

**Table 1.** Characteristics of the study population (n=155).

Characteristics			Survivors (n=155)
**Sex, n (%)**			
	Female		100 (64.5)
	Male		55 (35.5)
**Age at counseling (years)**			
	Median (IQR)		30 (24-39.5)
	Range		18-63
**Age at cancer diagnosis (years)**			
	Median (IQR)		12 ( 5-16)
	Range		0-35
**Primary cancer diagnosis** ^a^ **, n (%)**			
	Lymphomas		45 (29)
	Leukemias		42 (27.1)
	Brain tumors		34 (21.9)
	Sarcomas		19 (12.3)
	Embryonic tumors		7 (4.52)
	Rare tumors		8 (5.2)
Radiation, n (%)			95 (61.3)
Total body or skull irradiation, n (%)			61 (39.4)
Chemotherapy, n (%)			142 (91.6)
Stem cell transplantation, n (%)			25 (16.1)
Operation, n (%)			75 (48.4)
Recurrence or relapse			27 (17.4)
**Risk group, n (%)**			
	1		18 (11.6)
	2		42 (27.1)
	3		95 (61.3)
**Education level** ^a^ **, n(%)**			
	[Table-fn T1_FN1]No school degree		2 (1.3)
	Lower secondary education or less (ISCED ≤2)		14 (9)
	Upper secondary+ non-tertiary postsecondary education (ISCED 3‐4)		48 (30.9)
	Tertiary education (ISCED ≥5)		91 (58.7)
Previous lifestyle counseling, n (%)			82 (52.9)
**Desire for lifestyle counseling, n (%)**			
	Sports and nutrition		39 (25.2)
	Sports		27 (17.4)
	Nutrition		15 (9.7)
	None		74 (47.7)
Lifestyle counseling performed, n (%)			120 (77.4)
**Recommendations, n (%)**			
	Sports and nutrition		65 (54.2)
	Sports		43 (35.8)
	Nutrition		8 (6.7)
	None		4 (3.3)

a According to the International Standard Classification of Education (ISCED) [30].

### Needs Analysis and Lifestyle Counseling

Lifestyle counseling was provided to 120 survivors (response rate 77.4%) including 112 survivors with a need for counseling from either the physician’s, the sports scientist’s, or the CCS’ perspective (Table 2). Additionally, 8 CCS opted for lifestyle counseling after receiving further study information although they initially did not indicate the need for it. They were open to recommendations and the talk turned into counseling.

For 27 (17%) survivors, there was no need for counseling, while the remaining 8 (5%) survivors did not want counseling even though there was a need from either the physician’s or the sports scientist’s perspective.

**Figure 1.**
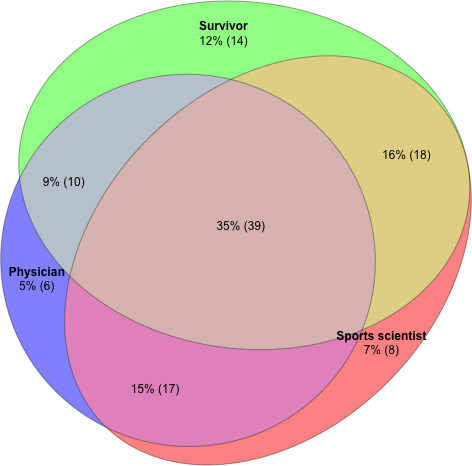
Overlap in the indication for lifestyle counseling based on the different perspectives of the physician, sports scientist, and childhood cancer survivor.

As the inclusion criteria for this study were based on the perspective of the survivor, the sports scientist, and the treating physician on who would benefit most from lifestyle counseling, we analyzed the need for counseling considering these three approaches. The indication for lifestyle counseling based on these different perspectives only matched in 39 (35%) CCS (Figure 1).

**Table 2.** Percentage of survivors (n=155) meeting the different inclusion criteria (activity [<150 min per week], BMI [≤18.5 or ≥30 kg/m^2^], diabetes mellitus [preexistent or HbA_1c_ ≥6.5%], hypercholesterolemia [≥5 mmol/L], and desire by patient).

Inclusion criteria	Survivors (n=155), n (%)
Activity	72 (46.5)
BMI	38 (24.5)
Diabetes mellitus	9 (5.8)
Hypercholesterolemia	43 (27.7)
Desire by patient	81 (52.3)

The median duration of the consultation was 25 (IQR 15-30) minutes, with a range of 10 to 60 minutes. Most frequently, CCS (65/120, 54%) were advised to change their lifestyle in both areas (diet and exercise). Furthermore, 51 out of the 120 (43%) CCS received recommendations for only exercise (43/120, 36%) or diet (8/120, 7%). There were 4 out of 120 (3%) CCS who, although interested in counseling, received no advice as they already met the lifestyle recommendations.

Almost all CCS (150/155, 97%) indicated that a general offer of lifestyle counseling would be useful in the context of LTFU.

### Follow-Up Interview

For motivational reasons and to check whether CCS were able to implement the recommendations, all CCS with counseling were asked if they wanted a second interview. A total of 16 (14%) CCS did not want, or as they already met the lifestyle recommendations, were not selected for a second interview. In 53 (44%) CCS, it could not be performed due to difficulties with the appointment arrangement outside of the regular LTFU interval. A second interview was performed in 51 (43%) CCS within 1 month. Of these, 49 (96%) CCS reported to have implemented the recommendations of the sports scientist in at least one of the areas, consisting of exercise and diet (Table 3), and 33 (65%) CCS reported to have improved their lifestyle in both areas, although only 21 (41%) CCS received counseling in both areas. In addition, 33 out of 51 (65%) CCS desired further support and counseling. Moreover, 50 CCS attending the follow-up appointment initially indicated that lifestyle counseling should be part of the LTFU care and confirmed this statement in the second consultation. One CCS indicated only in the follow-up appointment that lifestyle counseling would be useful.

**Table 3.** Characteristics of CCS^a^ who participated in the follow-up interview 1 month after lifestyle counseling.

Assessed at first counseling			Survivors (n=51)
Inclusion criteria (sports scientist), n (%)			36 (71)
Inclusion criteria (physician), n (%)			30 (59)
Desire of counseling (CCS), n (%)			32 (63)
**Characteristics at follow-up**			
	**Subjective benefit, n (%)**		
		No	2 (4)
		Yes	47 (92)
		Unclear	2 (4)
	**Implementation of the recommendations, n (%)**		
		Both	33 (65)
		Sports	8 (16)
		Nutrition	8 (16)
		No	2 (4)
	**Duration of the counseling (minutes)**		
		Median (IQR)	12 (10-19)
		Range	8‐40
	**Effect perceived, n (%)**		38 (75)
		Yes, weight loss	2 (4)
		Yes, quality of life	26 (51)
		Yes, both	8 (16)
		No	15 (29)

a CCS: childhood cancer survivors.

### Subgroup Analysis

Although the physicians’ and sports scientists’ inclusion criteria overlapped, 26 (17%) CCS need for counseling were only identified based on the sports scientist’s perspective (subgroup 1). A second interview was conducted with 13 of these CCS 1 month later, during which 12 (92%) CCS reported having benefited from counseling.

In addition, 14 (9%) CCS expressed a need for counseling from a personal point of view, although they did not meet the inclusion criteria from the physician’s or sports scientist’s perspective (subgroup 2). A second interview (recommended for 9 of these CCS) was conducted with 5 CCS, 4 (80%) of whom stated that they had benefited from the counseling.

Furthermore, 31 (20%) CCS did not express a need for counseling, although they met the inclusion criteria (subgroup 3). A total of 23 (74%) CCS agreed to receive lifestyle counseling. One month later, 15 of 16 (94%) participating CCS reported having benefited from counseling. These subgroups are also shown in more detail in Table 4.

**Table 4.** Characteristics of childhood cancer survivors in the subgroup analysis, subdivided into three subgroups.

Characteristics			Subgroup 1^a^ (n=26), n (%)	Subgroup 2^b^ (n=14), n (%)	Subgroup 3^c^ (n=31), n (%)	Descriptive *P* value^d^
**Sex**						.19
	Female		19 (73)	10 (71)	16 (52)	
	Male		7 (27)	4 (29)	15 (48)	
**Risk group**						.49
	1		3 (12)	3 (21)	2 (7)	
	2		7 (27)	4 (29)	6 (19)	
	3		16 (62)	7 (50)	23 (74)	
Indication for counseling by sports scientist			26 (100)	0 (0)	25 (81)	Not performed
Indication for counseling by physician			0 (0)	0 (0)	23 (74)	Not performed
Desire for counseling			18 (69)	14 (100)	0 (0)	Not performed
Counseling performed			25 (96)	13 (93)	23 (74)	.05
**Follow-up appointment^e^**						.59
	Performed		13 (50)	5 (36)	16 (52)	
	Not indicated by sports scientist or survivor		2 (8)	5 (36)	9 (29)	
	Not feasible		11 (42)	4 (29)	6 (19)	
**Implementation of the recommendations**						.35
	Both		7 (27)	3 (21)	9 (29)	
	Sports		4 (15)	0 (0)	4 (13)	
	Nutrition		1 (4)	1 (7)	3 (10)	
	No		1 (4)	1 (7)	0 (0)	
	No follow-up		0 (0)	9 (64)	15 (48)	

a Subgroup 1: survivors who have a need for counseling based only on the inclusion criteria of the sports scientist and not from the physician’s point of view.

b Subgroup 2: survivors who want lifestyle counseling but do not need it based on the criteria.

c Subgroup 3: survivors who need counseling based on the criteria but did not express a desire for counseling.

d Descriptive *P*-value from chi-square test (sex, follow-up appointment, implementation of the recommendations) and Fisher exact test (risk group, counseling performed).

e For follow-up appointment, we tested performed versus all other categories, and for implementation of the recommendations, we tested implementation of at least one recommendation versus all other categories.

## Discussion

### Principal Findings

Worldwide, as a result of advances in cancer treatment and diagnostics, the proportion of long-term cancer survivors is rapidly increasing [31]. As many of these survivors develop chronic health conditions later in life, prevention measures gain importance in follow-up care and management of these CCS in order to reduce long-term morbidity and maintain a good health-related quality of life. Regular physical activity can have a major impact on metabolic health and reduce risk factors for metabolic syndrome [11,17,32]. As shown in previous studies, long-term sequelae, like diabetes mellitus and obesity, occur more often in CCS than in the general population and contribute to elevated morbidity and mortality risk. This risk can be diminished by implementing a healthy lifestyle based on regular physical activity and a healthy diet [15,33].

In this study, indications for, as well as the feasibility of, regular lifestyle counseling in an unselected cohort of long-term CCS in a specialized LTFU care setting were prospectively evaluated. In addition, we aimed to identify CCS who could particularly benefit from counseling based on three different perspectives (physician’s, CCS’, and sports scientist’s view).

During the study, over 77% (120 out of 155) of the CCS received lifestyle counseling with a special focus on physical activity and nutrition. About 48% (n=74) of these CCS expressed no need for counseling initially, but were either interested in lifestyle recommendations or had indicators for counseling (based on metabolic risk constellations or reduced physical activity). Consequently, 39 CCS received lifestyle counseling who initially did not state a need for it.

Due to difficulties in contacting, only 51 CCS received a follow-up appointment. Almost all of these CCS (48/51, 94%) indicated that they benefited from lifestyle counseling which is in line with previous studies that highlighted the importance of lifestyle counseling in an unselected CCS cohort as it resulted in higher activity levels after seeing a health practitioner [32]. Considering different perspectives on who would benefit from counseling resulted in a high participation rate and satisfaction with the offer. However, the overlap between the indications for counseling was poor. We classified CCS into three distinct subgroups. Our findings demonstrated that lifestyle counseling can be beneficial for CCS, even if only one perspective recognizes the indication for it. This highlights the relevance of a multidisciplinary approach in LTFU care including sports scientists to support a healthy lifestyle among this cohort [34,35].

Furthermore, our results show that especially CCS with a high risk for late effects (risk group 3 with radiotherapy exposure, according to Gebauer et al [25]) are in higher need of lifestyle counseling. According to Rock et al [36], survivors of pediatric acute lymphoblastic leukemia have an increased risk of becoming overweight throughout their lives. Due to cancer treatment in childhood, like radiotherapy exposure, metabolic late effects such as overweight, obesity, and changes in body composition like an increase in fat mass are common in CCS. They could also be demonstrated in our cohort, with 46.5% (70/155) of the survivors being affected by metabolic disorders [9,37,38]. Cranial radiation often leads to endocrine disorders which in turn may result in overweight or obesity [38,39]. Of note, although the proportion of CCS with an indication for lifestyle counseling was high across all risk groups, CCS with the lowest risk for late effects (risk group 1 according to Gebauer et al [25]) appeared to be affected more often than CCS in risk group 2 (medium risk for late effects). This is most likely due to the small number of CCS in risk group 1 included in this study resulting in a selection bias. This finding should be verified in further studies with more participants representing the different risk groups (based on cancer treatment exposure) in a more balanced way. However, the study findings suggest that lifestyle counseling should be considered more strongly for individuals in higher-risk groups. Consequently, CCS in risk group 3, in particular, should be given serious consideration for regular lifestyle counseling as already proposed by Nathan et al [39].

The fact that almost all CCS (n=150, 97%) considered lifestyle counseling to be beneficial, and those who reported benefits from it in the follow-up appointment (47/51, 92%), support the idea of incorporating lifestyle counseling into regular LTFU care. In addition, 70% (36/51) of CCS receiving a second counseling stated a positive effect on weight or quality of life. These findings are consistent with previous studies that have shown high levels of acceptance and adherence to lifestyle counseling [10,33]. Zhang et al [15,40], suggest that it is crucial to educate CCS about weight management and healthy lifestyle as early as possible, but older CCS may require more lifestyle counseling. Moreover, men may have a greater need for lifestyle counseling (based on the presence of metabolic diseases or a sedentary lifestyle) as demonstrated in this study but often do not perceive this need. Furthermore, the study revealed that CCS with a normal weight who do not meet the inclusion criteria can still derive benefits from counseling. Additionally, there were 29 (19%) CCS with a prediabetic metabolic condition, who could benefit from counseling and should be included in future studies.

Given the constraints of limited resources, it is crucial to deliberate on the inclusion criteria for lifestyle counseling. As demonstrated in this study, factors such as the higher risk for late effects (risk group 3), based on treatment exposure, older age, male gender, and reduced physical activity, as well as established cardiovascular risk factors, such as the presence of obesity, diabetes mellitus, or hypercholesterolemia could serve as potential indicators for lifestyle counseling.

### Limitations and Strengths of the Study

Limitations of this study included heterogeneity within the study cohort such as predominance of female gender (n=100, 65%) and risk group 3 (n=95, 61%). This was a result of an unselected inclusion of every CCS receiving regular LTFU care in our specialized clinic, which renders comparison of different risk groups more difficult. A higher proportion of women in LTFU has also been observed in previous studies performed in our cohort, as well as in different LTFU cohorts, and was discussed as a consequence of an increased engagement in preventive health behavior in women compared with men [41,42].

In addition, 53% (n=82) of all CCS already received previous nutrition or sports counseling which may have had an impact on their lifestyle. However, the content of previous counseling was not recorded in a structured way in this study. Furthermore, in 53 (43%) CCS, a follow-up appointment could not be performed due to organizational difficulties. Some measurements were based solely on information provided by the CCS such as nutrition habits and weight loss after counseling and could not be validated clinically. For future research, it would be useful to assess data more objectively and after a longer period of time to better assess the long-term effect on metabolic parameters and activity. A follow-up after 1 month, in this context, is not sufficient to fully capture the impact of lifestyle counseling. However, in the CARE for CAYA study that offered lifestyle counseling to a predefined proportion of CCS, no significant effect of the intervention in a follow-up after 1 year could be demonstrated. As a consequence of these results, for this analysis, we intended to investigate the short-term effect of counseling assuming that the effects might be most pronounced during the first weeks [43].

The small number of CCS experiencing nutritional issues (n=15) may be attributed to the fact that the assessment of nutritional parameters relied solely on subjective questioning. Especially for the evaluation of activity time, it would have been useful to collect either objective data by accelerometry or the moderate and vigorous activity minutes in the follow-up counseling. The assessment of the quality of life did not involve the use of questionnaires; instead, CCS provided subjective responses.

However, to our knowledge, this is the first study to implement regular lifestyle counseling for every CCS in a specialized setting with many years of experience within LTFU care. We collected the data from an unselected cohort in a prospective manner, which allowed us to include all CCS receiving LTFU care and gain an overall understanding of their needs. For further research, it might be useful to analyze CCS after lifestyle counseling over a longer period to see whether they sustained a health-promoting lifestyle. Furthermore, it would be useful for future studies to promote a mobile health intervention for CCS that might increase adherence to adopting a healthier lifestyle [44].

### Conclusions

Lifestyle counseling is feasible and considered useful by most CCS. However, due to limited resources, counseling might not be available for every CCS during LTFU. Therefore, it is particularly important to identify CCS at risk for metabolic complications. Although early implementation of lifestyle changes is recommended in order to reduce long-term morbidity, as demonstrated in this study, older CCS, as well as male CCS, were especially in need of counseling. This group should be actively screened for established risk factors such as hypertension, diabetes mellitus, and hypercholesterolemia. Additionally, a more intensive cancer treatment exposure was confirmed as an independent risk factor for poor metabolic outcome and should prompt initiation of counseling. Consequently, as a healthy lifestyle including regular physical activity and a nutrient- and vitamin-rich diet can reduce survivors’ increased risk of developing metabolic diseases, it should be part of regular LTFU care for CCS.

## Supplementary material

10.2196/59614 Multimedia Appendix 1 Lifestyle counseling record sheet.

## Abbreviations

ASC: American Cancer Society
CCS: childhood cancer survivors
HbA_1c_: hemoglobin A_1c_
LTFU: long-term follow-up

## References

[1] Wellbrock M, Spix C, Ronckers CM (2023). Temporal patterns of childhood cancer survival 1991 to 2016: a nationwide register-study based on data from the German Childhood Cancer Registry. Int J Cancer.

[2] Erdmann F, Kaatsch P, Grabow D, Spix C German childhood cancer registry—annual report 2019 (1980-2018). https://www.kinderkrebsregister.de/fileadmin/kliniken/dkkr/pdf/jb/jb2019/Buch_DKKR_Jahresbericht_2019_komplett.pdf.

[3] Bhakta N, Liu Q, Ness KK (2017). The cumulative burden of surviving childhood cancer: an initial report from the St Jude Lifetime Cohort Study (SJLIFE). Lancet.

[4] Gebauer J, Higham C, Langer T, Denzer C, Brabant G (2019). Long-term endocrine and metabolic consequences of cancer treatment: a systematic review. Endocr Rev.

[5] Jones LW, Liu Q, Armstrong GT (2014). Exercise and risk of major cardiovascular events in adult survivors of childhood hodgkin lymphoma: a report from the childhood cancer survivor study. J Clin Oncol.

[6] Ehrhardt MJ, Leerink JM, Mulder RL (2023). Systematic review and updated recommendations for cardiomyopathy surveillance for survivors of childhood, adolescent, and young adult cancer from the International Late Effects of Childhood Cancer Guideline Harmonization Group. Lancet Oncol.

[7] Brinkman TM, Recklitis CJ, Michel G, Grootenhuis MA, Klosky JL (2018). Psychological symptoms, social outcomes, socioeconomic attainment, and health behaviors among survivors of childhood cancer: current state of the literature. JCO.

[8] van Deuren S, Boonstra A, van Dulmen-den Broeder E, Blijlevens N, Knoop H, Loonen J (2020). Severe fatigue after treatment for childhood cancer. Cochrane Database Syst Rev.

[9] Smith WA, Li C, Nottage KA (2014). Lifestyle and metabolic syndrome in adult survivors of childhood cancer: a report from the St. Jude Lifetime Cohort Study. Cancer.

[10] de Lima Melo B, Vieira DCA, de Oliveira GC (2023). Adherence to healthy lifestyle recommendations in Brazilian cancer survivors. J Cancer Surviv.

[11] Rock CL, Thomson CA, Sullivan KR (2022). American Cancer Society nutrition and physical activity guideline for cancer survivors. CA Cancer J Clin.

[12] Javalkar K, Huang Y, Lyon SM (2023). Clinical response to lifestyle counseling for dyslipidemia and elevated blood pressure in childhood cancer survivors. Pediatr Blood Cancer.

[13] Clinton SK, Giovannucci EL, Hursting SD (2020). The World Cancer Research Fund/American Institute for Cancer Research third expert report on diet, nutrition, physical activity, and cancer: impact and future directions. J Nutr.

[14] Oeffinger KC, Mertens AC, Sklar CA (2006). Chronic health conditions in adult survivors of childhood cancer. N Engl J Med.

[15] Zhang FF, Kelly MJ, Must A (2017). Early nutrition and physical activity interventions in childhood cancer survivors. Curr Obes Rep.

[16] Hammoud RA, Mulrooney DA, Rhea IB (2024). Modifiable cardiometabolic risk factors in survivors of childhood cancer: *JACC: CardioOncology* state-of-the-art review. JACC CardioOncol.

[17] Ness KK, Leisenring WM, Huang S (2009). Predictors of inactive lifestyle among adult survivors of childhood cancer: a report from the Childhood Cancer Survivor Study. Cancer.

[18] Devries MC, Jakobi JM (2021). Importance of considering sex and gender in exercise and nutrition research. Appl Physiol Nutr Metab.

[19] de Haas EC, Oosting SF, Lefrandt JD, Wolffenbuttel BH, Sleijfer DT, Gietema JA (2010). The metabolic syndrome in cancer survivors. Lancet Oncol.

[20] Danielzik S, Czerwinski-Mast M, Langnäse K, Dilba B, Müller MJ (2004). Parental overweight, socioeconomic status and high birth weight are the major determinants of overweight and obesity in 5-7 y-old children: baseline data of the Kiel Obesity Prevention Study (KOPS). Int J Obes.

[21] Jones LW, Demark-Wahnefried W (2006). Diet, exercise, and complementary therapies after primary treatment for cancer. Lancet Oncol.

[22] Dimitri P, Joshi K, Jones N, Moving Medicine for Children Working Group (2020). Moving more: physical activity and its positive effects on long term conditions in children and young people. Arch Dis Child.

[23] Gebauer J, Rieken S, Schuster S (2018). Multidisciplinary late effects clinics for childhood cancer survivors in Germany—a two-center study. Oncol Res Treat.

[24] Salchow J, Mann J, Koch B (2020). Comprehensive assessments and related interventions to enhance the long-term outcomes of child, adolescent and young adult cancer survivors—presentation of the CARE for CAYA-Program study protocol and associated literature review. BMC Cancer.

[25] Gebauer J, Baust K, Bardi E (2020). Guidelines for long-term follow-up after childhood cancer: practical implications for the daily work. Oncol Res Treat.

[26] Zand A, Ibrahim K, Patham B (2018). Prediabetes: why should we care?. Methodist Debakey Cardiovasc J.

[27] Frost AP, Norman Giest T, Ruta AA, Snow TK, Millard-Stafford M (2017). Limitations of body mass index for counseling individuals with unilateral lower extremity amputation. Prosthet Orthot Int.

[28] Schmitz KH, Courneya KS, Matthews C (2010). American College of Sports Medicine roundtable on exercise guidelines for cancer survivors. Med Sci Sports Exerc.

[29] Arends J, Bertz H, Bischoff S (2015). S3-Leitline der Deutschen Gesellschaft für Ernährungsmedizin e. V. (DGEM) in Kooperation mit der Deutschen Gesellschaft für Hämatologie und Onkologie e. V. (DGHO), der Arbeitsgemeinschaft „Supportive Maßnahmen in der Onkologie, Rehabilitation und Sozialmedizin“ der Deutschen Krebsgesellschaft (ASORS) und der Österreichischen Arbeitsgemeinschaft für klinische Ernährung (AKE) [Article in German]. Akt Ernahrungsmed.

[30] Mogensen H, Tettamanti G, Frederiksen LE (2024). Educational attainment in survivors of childhood cancer in Denmark, Finland, and Sweden. Br J Cancer.

[31] Hudson MM, Bhatia S, Casillas J, Landier W, Section on Hematology/Oncology, Children’s Oncology Group, American Society of Pediatric Hematology/Oncology (2021). Long-term follow-upcare for childhood, adolescent, and young adult cancer survivors. Pediatrics.

[32] Myers J, Kokkinos P, Nyelin E (2019). Physical activity, cardiorespiratory fitness, and the metabolic syndrome. Nutrients.

[33] Tarasenko YN, Miller EA, Chen C, Schoenberg NE (2017). Physical activity levels and counseling by health care providers in cancer survivors. Prev Med.

[34] Huang YJ, Lee SL, Wu LM (2021). Health-promoting lifestyle and Its predictors in adolescent survivors of childhood cancer. J Pediatr Oncol Nurs.

[35] Bouwman E, Pluijm SMF, Stollman I (2023). Healthcare professionals’ perceived barriers and facilitators of health behavior support provision: a qualitative study. Cancer Med.

[36] Rock CL, Doyle C, Demark-Wahnefried W (2012). Nutrition and physical activity guidelines for cancer survivors. CA Cancer J Clin.

[37] Guolla L, Morrison KM, Barr RD (2021). Adiposity in survivors of cancer in childhood: how is it measured and why does it matter?. J Pediatr Hematol Oncol.

[38] Pradhan KR, Chen Y, Moustoufi-Moab S (2019). Endocrine and metabolic disorders in survivors of childhood cancers and health-related quality of life and physical activity. J Clin Endocrinol Metab.

[39] Nathan PC, Ford JS, Henderson TO (2009). Health behaviors, medical care, and interventions to promote healthy living in the childhood cancer survivor study cohort. J Clin Oncol.

[40] Zhang FF, Parsons SK (2015). Obesity in childhood cancer survivors: call for early weight management. Adv Nutr.

[41] Sleimann M, Balcerek M, Cytera C (2023). Implementation of a clinical long-term follow-up database for adult childhood cancer survivors in Germany: a feasibility study at two specialised late effects clinics. J Cancer Res Clin Oncol.

[42] Tinner EME, Dogan O, Boesing M (2024). Characteristics and feedback of adult survivors of childhood cancer seen in Swiss comprehensive follow-up clinics led by general internists: a prospective cohort study. BMJ Open.

[43] von Grundherr J, Elmers S, Koch B (2024). A multimodal lifestyle psychosocial survivorship program in young cancer survivors: the CARE for CAYA program—a randomized clinical trial embedded in a longitudinal cohort study. JAMA Netw Open.

[44] Kelley MM, Kue J, Brophy L (2021). Mobile health applications, cancer survivors, and lifestyle modification: an integrative review. Comput Inform Nurs.

[45] Richter F, Kronziel LL, König IR, Langer T, Gebauer J (2023). High incidence of metabolic diseases and sedentary lifestyle underline the need for regular counseling in LTFU for CCS- results from a monocentric prospective study. Research Square.

