# Supplementary material for: Implementation of Regular Lifestyle Counseling During Long-Term Follow-Up Care of Childhood Cancer Survivors: Monocentric Prospective Study
Source: JMIR Cancer. 2024 Dec 26;10:e59614. doi: 10.2196/59614 (PMC11694152; doi:10.2196/59614)
Supplement: Multimedia Appendix 1 [file cancer-v10-e59614-s001.docx]

**Lifestyle counseling – record sheet**

Basic data:

| Name: | Date: | | Oncological disease: | |
| --- | --- | --- | --- | --- |
| Age: | Age at first diagnosis: | | Irradiation: Yes / No | |
| Chemotherapy: Yes / No | SCT: Yes/no | | Recurrence: Yes / No | |
| Operation: | Educational Qualifications: | | | |
| Occupation: | | Employment relationship: FT – PT - JS | | Weekly working hours: |

SCT; stem cell transplantation, FT; full time, PT; part time, JS; job seeking.

Needs analysis:

| Height: | Weight: | BMI: |
| --- | --- | --- |
| Diabetes mellitus: Yes / No | Hypercholesterolemia: Yes / No |  |
| Min Moderate/week: | Min Intensive/week: |  |

Have you had any previous sports or nutrition counseling? Yes No

If so, to what extent?

Do you think a general offer of sports/nutrition counseling in the context of LTFU would be useful?

Yes No

Do you think you need advice in the area(s) of sports and/or nutrition?

- Yes, both areas

Why:

- Yes, sport
- Yes, nutrition
- No, not desired

From your point of view, would you like to change your behavior in terms of acticity or nutrition?

Yes, which area: No, why not?

Consulting content: Consultation duration:

**Follow Up Appointment**

**Follow up**

1. Were you able to implement recommendations?

- Yes, which:
- No, why not:
- Partially, which:

1. Have you noticed any changes?

Positive:

Negative:

1. Did you benefit from the counseling? Yes No

1. Would you like to receive further support? Yes No
2. Do you think a general offer of lifestyle counseling in the context of LTFU would be useful?

Yes No

Consultation duration:

Date:
